# Effects of *BrMYC2/3/4* on Plant Development, Glucosinolate Metabolism, and *Sclerotinia sclerotiorum* Resistance in Transgenic *Arabidopsis thaliana*

**DOI:** 10.3389/fpls.2021.707054

**Published:** 2021-09-03

**Authors:** Zhiyan Teng, Weiwei Zheng, Youjian Yu, Seung-Beom Hong, Zhujun Zhu, Yunxiang Zang

**Affiliations:** ^1^Collaborative Innovation Center for Efficient and Green Production of Agriculture in Mountainous Areas of Zhejiang Province, College of Horticulture Science, Zhejiang A&F University, Hangzhou, China; ^2^Department of Biotechnology, University of Houston Clear Lake, Houston, TX, United States

**Keywords:** *BrMYC2/3/4*, plant development, glucosinolate, *Sclerotinia sclerotiorum*, disease resistance

## Abstract

MYC2/3/4, known as a basic helix–loop–helix (bHLH) transcription factor, directly activate the genes involved in diverse plant development and secondary metabolites biosynthesis. In this study, we identified and cloned five *MYC* paralogs (*BrMYC2*/*3-1*/*3-2*/*4-1*/*4-2*) from Chinese cabbage (*Brassica rapa* ssp. *pekinensis*). *In-silico* analyses for the physicochemical properties suggested that BrMYC2/3-1/3-2/4-2/4-3 are unstable hydrophobic and acidic proteins, while BrMYC4-1 is an unstable hydrophobic and basic protein. BrMYC2/3/4 belong to the bHLH superfamily and are closely related to AthMYC2/3/4 orthologs that mediate the regulation of various secondary metabolites. It was demonstrated that BrMYC2/3/4-GFP fusion protein localized in the nucleus and expression levels of five *BrMYC2/3/4* homologous genes all elevated relative to control (Ctrl). When expressed in *Arabidopsis* under the control of 35S promoter, each of the *BrMYC2/3-1/3-2/4-1/4-2* transgenes differentially influenced root and shoot elongation, vegetative phase change, flowering time, plant height and tiller number after flowering, and seed production. Despite the variation of phenotypes between the transgenic lines, all the lines except for *BrMYC4-2* exhibited shorter seed length, less seed weight, higher accumulation of glucosinolates (GSs), and resistance to *Sclerotinia sclerotiorum* than Ctrl. Notably, *BrMYC2* overexpression (OE) line significantly reduced the lengths of root and hypocotyl, seed length, and weight, along with faster bolting time and strikingly higher accumulation of total GSs. Accumulation of GSs at the highest levels in the *BrMYC2*^*OE*^ line conferred the highest resistance to *S. sclerotiorum*. Unlike *BrMYC3*^*OE*^ and *BrMYC4*^*OE*^, *BrMYC2*^*OE*^ stimulated the growth of plant height after fluorescence. The results of this study point to the *BrMYC2* overexpression that may provide a beneficial effect on plant growth and development *via* plant resistance to the fungal pathogen.

## Introduction

Myelocytomatosis proteins (MYCs) are key transcription factors (TFs) in the response pathway of jasmonic acid (JA) in plants (Xie et al., 2020). Jasmonates (JAs) and its derivatives of JA methyl ester and amino acid conjugates (Per et al., 2018; Stella de Freitas et al., 2019) are well-known phytohormones connecting both developmental signals and environmental stresses to coordinately regulate diverse metabolic processes involved in plant growth and development (Song et al., 2017). Examples are seed development (Cao et al., 2016), root growth (To et al., 2019), shoot elongation (To et al., 2019), trichome development (del Rosario Cappellari et al., 2019), and fertility (Schubert et al., 2019). MYC2 (bHLH006), MYC3 (bHLH005), and MYC4 (bHLH004) belong to the family of the IIIe subgroup basic helix–loop–helix (bHLH) and contain a conserved domain of bHLH (Zhang et al., 2018, 2020; Wasternack and Strnad, 2019). MYC2/3/4 interact with F-box protein CORONATINE INSENSITIVE1 (COI1) (Major et al., 2017) and JA ZIM-domains (JAZs) (Garrido-Bigotes et al., 2018) to mediate diverse JA response, such as root growth inhibition (Yang et al., 2017), stamen development (Zhuo et al., 2020), leaf senescence (Xu et al., 2018), glucosinolates (GSs) biosynthesis (Costarelli et al., 2020), defenses against insect attack (Chen et al., 2021), and necrotrophic fungi (Tiwari, 2018).

Glucosinolates are a group of secondary metabolites found in *Brassicaceae* family and are composed of β-D-thioglucose and sulfonated oxime moieties (Tiwari, 2018). They have received considerable interest because of their commercial properties of anti-cancer agents (Arumugam and Razis, 2018), bio-pesticides (Malka and Cheng, 2017), and flavor condiment (Burow et al., 2015). They are typically classified as aliphatic GS (from alanine, valine, leucine, isoleucine, or methionine), aromatic GS (from phenylalanine or tyrosine), or indole GS (from tryptophan) on the basis of their modified side chains (R) derived from amino acids through a long chain lengthening process and hydroxylation or oxidation (Rahimi and Rahmanpour, 2020). Biosynthesis and metabolism of GSs are a complex process that is finely regulated by a variety of trans-acting regulatory factors; these include eight TFs of v-myb avian myeloblastosis viral oncogene homolog (MYB) family (Frerigmann and Gigolashvili, 2014; Zhang et al., 2015; Seo and Kim, 2017; Coleto et al., 2021), three TFs of MYC family (MYC2, MYC3, and MYC4) (Schweizer et al., 2013), and other TFs, such as Dof1.1, IQD1 (Kim et al., 2014), and ATR1 (Kai et al., 2011). MYC2, MYC3, and MYC4 are the main TFs regulating indole GSs (IGSs) biosynthesis, since those mutants significantly reduced levels of IGSs (Schweizer et al., 2013; Frerigmann et al., 2014). Non-methionine aliphatic GSs (AGSs) are present in all the *Brassicaceae* families, and the indole pathway is thought to have originated from a whole-genome duplication event (Mitreiter and Gigolashvili, 2021).

Chinese cabbage (*Brassica rapa* ssp. *pekinensis*) is an economically important leafy vegetable that is largely cultivated around the world, especially in Korea, Japan, and China, and its genome sequencing has been completed (Park et al., 2019). Due to the evolutionary relatedness, most of the genes in Chinese cabbage contain more than one ortholog of *Arabidopsis* (Wang et al., 2011). GSs and their hydrolysis products are known to play essential roles in plant–insects and plant–microbes' interactions (Pastorczyk et al., 2020; Ji et al., 2021). For example, isothiocyanates (ITCs), 4-hydroxyglucobrassicin (4-OHGBC), and 4-methoxyglucobrassicin (4MeGBC) are involved in plant resistance against pests (Pfalz et al., 2019; Döring and Ulber, 2020; Shukla and Beran, 2020), as well as plant innate immunity (Clay et al., 2009; Pangesti et al., 2016; Chen et al., 2020). ITCs are the main volatile compounds resulting from the hydrolysis of GSs; 3-butenyl-(55.6–71.8%) and 5-methylsulfinylpentyl-ITC (0–9.5%) originate from gluconapin (GNA) and glucoalyssin (GAS), respectively (BlaŽević et al., 2011). Anti-microbial activity of such volatile compounds was found to inhibit a wide range of bacteria and fungi (BlaŽević et al., 2019). Indole-3-carbinol (I3C), a hydrolysate of 4MeGBC, aides in guiding the oviposition of specific insects in *Cruciferous* vegetables (Katz and Chamovitz, 2017).

Evolutionary data suggest that Chinese cabbage is closely related to *A. thaliana* and that it underwent a whole-genome triplication since it has diverged from *Arabidopsis thaliana* (Wang et al., 2011). There are one, two, and three copies of *BrMYC2/3/4* family genes in Chinese cabbage, respectively. Functions of *MYC2/3/4* orthologs have been mainly investigated in *A. thaliana*, and they showed a functional divergence (Schweizer et al., 2013). However, the functions and regulatory roles of multiple paralogous copies of *BrMYC2/3/4* family genes have remained unresolved.

In the current study, we identified, cloned five *MYC2/3/4* paralogs (*BrMYC2, BrMYC3-1, BrMYC3-2, BrMYC4-1*, and *BrMYC4-2*) in Chinese cabbage, and conducted *in-silico* comparative studies on physicochemical properties, domains, and evolutionary relationships among the deduced BrMYC2/3/4 proteins. In order to study the phenotypes conferred by expression of BrMYC2/3/4, each of the cloned coding sequence of five *MYC2/3/4* paralogs was introduced into a binary vector pCAMBIA2302, and each chimeric transgene expression construct was transformed into *Arabidopsis*. Subsequently, T2 lines showing 3:1 segregation ratio of selectable marker gene were selected, and T3 lines were used to analyze various phenotypes of plants at juvenile and adult stage, GSs contents, and resistance to white mold caused by necrotrophic *Sclerotinia sclerotiorum*.

## Materials and Methods

### Identification of *B. Rapa MYC2/3/4* Genes

Gene IDs for *BrMYC2/3/4* genes of Chinese cabbage, BraA05g023030.3C (*BrMYC2*), BraA09g022310.3C (*BrMYC3-1*), BraA06g041690.3C (*BrMYC3-2*), BraA01g009460.3C (*BrMYC4-1*), BraA01g009470.3C (*BrMYC4-2*), and BraA08g000150.3C (*BrMYC4-3*), were retrieved from *Brassica* database annotations (http://brassicadb.cn/#/Annotations/). Coding sequences of the retrieved *BrMYC2/3/4* genes were obtained from *Brassica* database gene sequence (http://brassicadb.cn/#/GeneSequence/).

### *In-silico* Analyses

The amino sequences deduced from coding sequences were used to predict the functional properties of BrMYC2/3/4 proteins. Physiochemical properties were inferred using ProtParam software (http://web.expacy.org/protparam/). The percentages of sequence identities among and between MYC2/3/4 in terms of coding sequence and deduced amino acid sequence were analyzed using DNAMAN software version 6.0 (Lynnon Biosoft, Montreal, Canada). The domains were predicted using the conserved domain database (CDD, https://www.ncbi.nlm.nih.gov/Structure/cdd/wrpsb.cgi), which has a domain prediction tool provided by the National Center for Biotechnology Information (NCBI, US National Library of Medicine). Structural and evolutionary analyses of *MYC2/3/4* coding sequences in the U's triangle species were conducted with homologous sequences on *Brassica* database (http://brassicadb.cn/#/BLAST/) according to the neighbor-joining method (Tarahomi et al., 2020) using molecular evolutionary genetics analysis software (MEGA7 software) (Kumar et al., 2016). The main parameter settings were distance model, *p*-distance method (Sáez-López et al., 2019), gene tree robustness detection, bootstrap test (1,000 replicates) (Elateek et al., 2020), processing of gap missing data, and deletion between pairs.

### Expression Constructs

All cloning experiments were carried out through homologous recombination using the One Step Cloning Kit (Vazyme, Nanjing, China). The coding sequences of *BrMYC2, BrMYC3-1, BrMYC3-2, BrMYC4-1*, and *BrMYC4-2* were put under control of the constitutive CaMV 35S promoter in the binary vector pCAMBIA2302 with green fluorescent protein (GFP). The integrity of the cloned coding sequence was verified by colony PCR, followed by DNA sequencing.

### Plant Materials and Growth Conditions

*Arabidopsis* ecotype Col-0 was used as the wild-type for all experiments. Plant seeds were sown in a mixture of grass charcoal soil and vermiculite as 1:1 ratio and cultivated in a greenhouse for 2 months under well-controlled conditions of 65% relative humidity and 600 μmol·m^−2^·s^−1^ maximum light intensity. The photoperiod and temperature were 16/8 h (light/dark) and 21/19°C, respectively, in day/night.

### Plant Transformation and Transgenic Plants Selection

Recombinant plasmids (pCAMBIA2302, p2302MYC2, p2302MYC3-1, p2302MYC3-2, p2302MYC4-1, and p2302MYC4-2) were introduced into the *Agrobacterium tumefaciens* strain GV3101 (Shanghai Weidi Biotechnology Co, Ltd, China). *Agrobacterium*-mediated transformation was performed to produce T0 transgenic lines using the floral dip method (Van Eck, 2018). Overexpression (OE) transgenic lines obtained were named as Ctrl, *BrMYC2*^*OE*^, *BrMYC3-1*^*OE*^, *BrMYC3-2*^*OE*^, *BrMYC4-1*^*OE*^, and *BrMYC4-2*^*OE*^, respectively.

*Arabidopsis* seeds from T0 lines were first surface sterilized using 75% (v/v) ethanol for 90 s and then washed three times with double-distilled water (ddH_2_O). The seeds were further sterilized in 10% bleach for 10 min and washed three times with ddH_2_O. They were subsequently sown onto 1 × MS medium (Phyto Technology Laboratories, Shawnee Mission, KS, USA) supplemented with 3% sucrose (pH 5.8 and 0.8% (w/v) plant tissue culture agar) and 50 μg·ml^−1^ kanamycin (Phyto Technology Laboratories, Shawnee Mission, KS, USA). Seedlings were chilled at 4°C for 2 days and transferred to a growth room with controlled conditions (25°C, 16:8 h, light:dark regime) grown vertically for 4 days. Healthy seedlings were then transferred to the soil and cultivated in a greenhouse with controlled conditions as described above. Homozygous plants were selected at the T3 generation based on the 3:1 segregation ratio of T2 line in the presence of kanamycin.

In order to observe GFP fluorescence, 5-day-old kanamycin resistant seedlings were used under a confocal laser scanning microscope, Olympus FluoView FV10i (Olympus, Tokyo, Japan). Images were recorded and contrast-enhanced using ImageJ software (ImageJ, 1.47v, NIH, Bethesda, USA). Some seedlings were subjected to plasmolysis in 1 M mannitol for 5 min (Xiao et al., 2014). GFP fluorescence was detected using a 488-nm excitation laser and 525/50-nm emission filter.

Leaf samples obtained from different T3 transgenic lines were harvested, frozen in liquid nitrogen, and stored at −80°C until later use.

### Semi-quantitative RT-PCR for Detection of Gene Expression Levels

Semi-quantitative RT-PCR to detect RNAs was carried out using total RNA isolated from 8-day-old seedlings. Total RNAs were isolated using the TRIzol reagent kit (Invitrogen) and treated with RNase-free DNase to remove any genomic DNA contaminants. All RNA samples were quantified using a NanoDrop 2000 spectrophotometer (Thermo Fisher Scientific Inc., Waltham, MA, USA) and were adjusted to the same concentration with diethylpyrocarbonate-treated water. Complementary DNA (cDNA) was synthesized using the PrimeScript™ II 1st Strand cDNA Synthesis Kit (Takara Bio, Inc, Otsu, Japan) following the protocol of the manufacturer using an oligo dT primer and 1 μg of total RNA. PCR was then performed using gene-specific primers and KOD Plus Neo (Toyobo, Osaka, Japan). The reaction was initiated by predenaturating at 94°C for 2 min, followed by 35 cycles of denaturation (98°C for 10 s), annealing (50°C for 30 s), and extension (68°C for 30 s), and was terminated with a final extension of 10 min at 68°C. The amplification products were analyzed by 1.2% agarose gel electrophoresis.

*AtActin2*, a housekeeping gene of *A. thaliana*, was used as an internal control to adjust the amount of cDNA template for PCR. Stably expressed *AtActin2* (AT3G18780) was used for normalization. *MYC2/3/4* and *Atactin2* primers are shown in Supplementary Table 1. Gel images were quantified using Gelpro 3.2 software (Media Cybernetics, Inc., Rockville, MD, USA). Three biological replicates were carried out for each sample.

### Seed Production Evaluations

In order to determine the seed yield, 30 T3 lines were taken from each genotype. After the siliques matured, the first five incompletely developed siliques were removed from the main inflorescence axis as described before (Jiang et al., 2020). The first intact siliques were then used to assess the number of seeds per silique. The mature siliques were tiled on A4 paper, and the carpel wall was removed with a dissecting needle, photographed with a Leica stereomicroscope (MZ16FA, Leica, Germany) to count the seed number per silique. The remaining siliques were allowed to mature, and seeds from the siliques located on the basis of a major inflorescence were selected for observation. Approximately 2,000 mature seeds from Ctrl and *BrMYC2/3-1/3-2/4-1/4-2*^*OE*^ of each line were randomly selected, observed, and photographed using a Leica stereomicroscope. The length and width of seeds were estimated using ImageJ software. Seed weight was measured using an electronic scale. Data points represent the average of 300 biological replicates.

### Plant Phase Change Evaluations

To evaluate the phase change among different genotypes, ~30 seedlings were randomly selected from each T3 line, transferred to an autoclaved mixture of grass charcoal soil and vermiculite a 1:1 ratio, and cultivated in the greenhouse for about 2–3 weeks. In order to assess whether the juvenile seedlings have reached the mature stage, abaxial trichomes as a hallmark of vegetative phase change were scored with a Leica stereomicroscope. For leaf shape analysis, fully expanded leaves were removed, attached to a cardboard with double-sided tape, flattened with transparent tape, and then scanned using the Epson V700 Professional scanner (Epson, Suwa, Japan). The bolting time and rosette leaf number at bolting time were recorded to determine the flowering time in *A. thaliana* (Xing et al., 2018). Plant height and tiller number were determined just after flowering. Pictures were taken from the plant adjacent to a ruler that was used to calibrate the ImageJ software for precise measurement of distance. Maximum height (from the growing point to the highest leaf tip as depicted by the vertical line) was estimated from the silhouette of *A. thaliana* differing in size. Measurements were obtained from 12 independent biological replicates.

### GSs Contents Determination

The leaves of 8-week-old homozygous T3 generation transgenic *A. thaliana* lines were used for determining GSs contents. Six seedlings were randomly selected from one transgenic line. GSs were essentially quantified as previously described (Zang et al., 2015). High-performance liquid chromatography (HPLC) analysis was performed using an Agilent1200 system (Agilent Technologies, Inc., Santa Clara, USA) with a C18 reverse-phase column (250 mm × 4 mm, 5 μm, Bischoff, Germany). Chromatography was performed over 60 min at a flow rate of 1 ml·min^−1^ in the following order: 100% H_2_O (2 min), a linear gradient of 0–20% ACN (32 min), 20% ACN (6 min), followed by 20–100% ACN (5 min), and 0% ACN before injecting the next sample. Eluents were monitored with a UV detector at 229 nm. Three biological and three technical replicates were performed.

### Antifungal Activity Bioassay

Lyophilized leaf powder of each transgenic *A. thaliana* line was used to study the effect of different GSs on the visible growth of *S. sclerotiorum*. Anti-fungal activity was essentially assayed as previously described with minor modifications (Kelemu et al., 2004). *S. sclerotiorum* was preserved at 4°C and then reactivated in a Petri dish containing potato dextrose agar (PDA) medium (Becton Dickinson, Columbia, MD). The mycelium was inoculated into the center using a 5 mm puncher; the Petri dishes were then incubated at 22°C for 72 h to provide actively growing mycelium for subsequent experiments. New marginal hyphae were excised with a puncher from the Petri dishes. Filter paper disks of 10 mm in diameter were then placed onto the surface of PDA. Each filter-paper disk received 25 mg lyophilized powder and 100 μl ddH_2_O, along with 100 μl ddH_2_O only as a control. None of the three filter paper disks was a PDA Petri dish, and each of the three Petri dishes was a biological repeat. The filter-paper disks carrying lyophilized powder and *S. sclerotiorum* were incubated at 22°C. Fungal growth was assessed by observing visible mycelium growth and sclerotia number per disk after 72 h of incubation. Three technical replicates were performed.

### Statistical Analysis

The data were evaluated using analysis of variance to determine statistical significance and were represented as means ± SD, as calculated by SPSS20.0 analysis software (IBM, Chicago, IL, USA). A difference was considered significant at the 95% confidence level (*p* < 0.05). All photographs were taken with a Nikon D5300 camera (Nikon Corporation, Tokyo, Japan) and edited in ImageJ. Graphs were plotted using Excel 2019 (Microsoft, Redmond, WA, USA) and figures were assembled using Microsoft PowerPoint.

## Results

### Structural Analyses of BrMYC2/3/4

Analysis of physicochemical properties showed that BrMYC2 consist of 605 amino acid residues, with a relative molecular mass of 65,848.54 and theoretical isoelectric point (pI) of 5.21, which suggests an acidic protein. Total average hydrophilicity and instability index are −0.548 and 45.84, respectively. Based on these values, BrMYC2 is an unstable hydrophobic and protein. BrMYC3-1 and BrMYC3-2 consist of 563 and 580 amino acid residues, with relative molecular masses of 61,940.76 and 63512.36, theoretical pI of 5.05 and 5.15, respectively. Total average hydrophilicity and instability index of BrMYC3-1 and BrMYC3-2 are −0.653 and −0.683, and 47.76 and 47.46, respectively. All these implicate that both BrMYC3-1 and BrMYC3-2 are unstable hydrophobic and acidic proteins. BrMYC4-1, BrMYC4-2, and BrMYC4-3 have 230, 297, and 60 amino acid residues with their relative molecular masses of 25,793.50, 31,956.93, and 6,509.31, respectively. Their theoretical pIs are 8.39, 4.66, and 5.05, respectively, suggesting that BrMYC4-1 is a basic protein, whereas BrMYC4-2 and BrMYC4-3 are acidic proteins. Total average hydrophilicity and instability index of BrMYC4-1, BrMYC4-2, and BrMYC4-3 are −0.647, −0.511, and −0.013 and 37.35, 46.81, and 39.05, respectively, indicating that BrMYC4-1, BrMYC4-2, and BrMYC4-3 are all hydrophobic and unstable proteins. In addition, the percentage of *BrMYC2/3/4* coding sequence identities was 55.40%, the percentage of *BrMYC2/3/4* deduced amino acid sequence identities was 47.40%. *MYC2/3/4* coding sequence and deduced amino acid sequence in *B. rapa* share 18.69–81.53% and 5.74–84.82% sequence identity with the *A. thaliana* orthologs, respectively.

### Domain Prediction of BrMYC2/3/4

**Figure 1:**
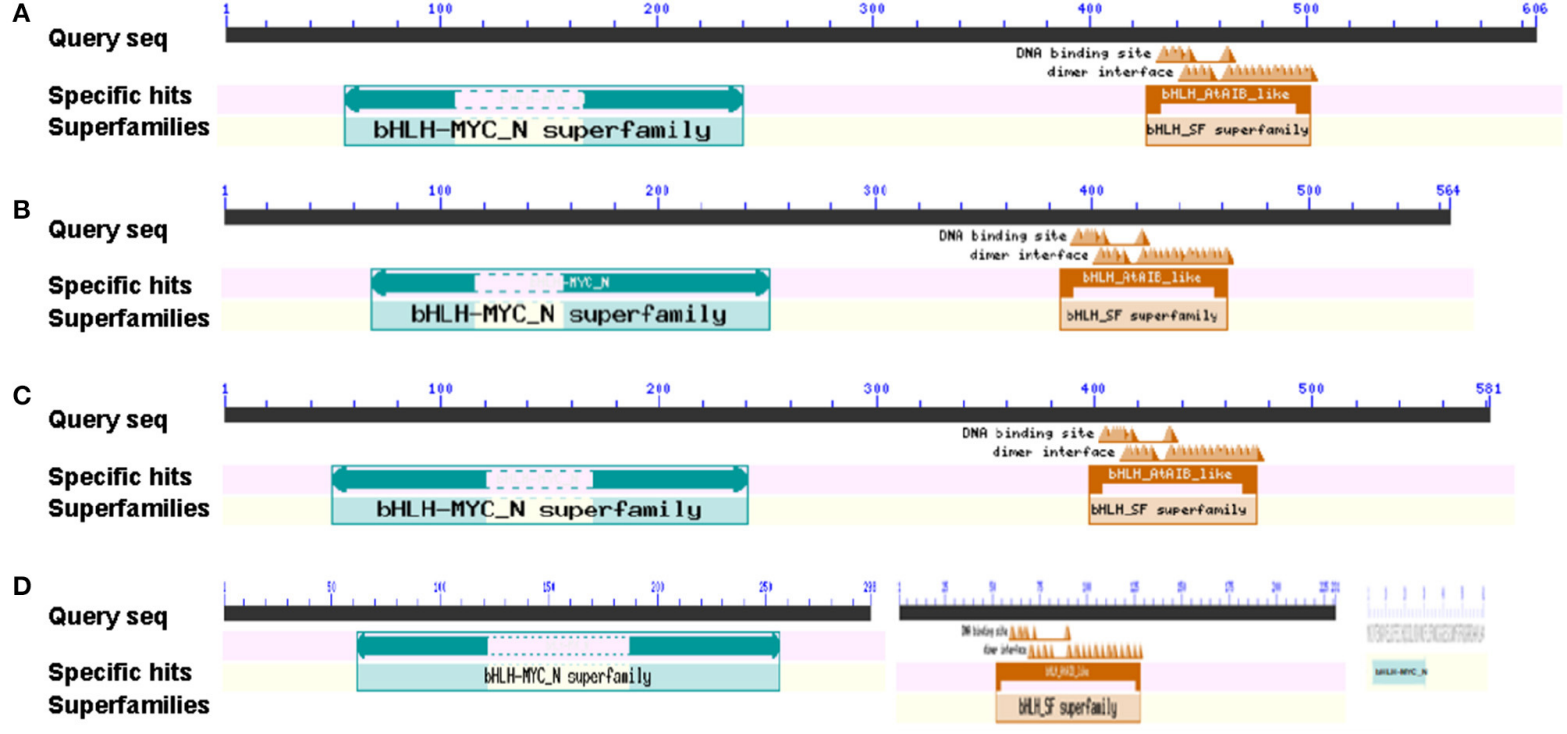
Putative *BrMYC2/3/4* DNA-binding domains in Chinese cabbage. **(A)** *BrMYC2* DNA-binding domains. **(B)** *BrMYC3-1* DNA-binding domains. **(C)** *BrMYC3-2* DNA-binding domains. **(D)** The DNA-binding domains illustrated; from left to right are *BrMYC4-2, BrMYC4-1*, and *BrMYC4-3*.

The protein domains encoded by BrMYC2/3/4 coding sequences were predicted using CDD, an NCBI online domain analysis software. The results showed that BrMYC2, BrMYC3-1, BrMYC3-2, and BrMYC4-2 have a conserved domain named bHLH-MYC_N superfamily; BrMYC2, BrMYC3-1, BrMYC3-2, and BrMYC4-1 have a conserved domain named bHLH_AtABA-inducible bHLH-TYPE_like in the C-terminal region; and BrMYC4-3 contains a single domain named bHLH-MYC_N (Figure 1). A bHLH_AtAIB_like is the bHLH-type domain found in AIB and MYC proteins (MYC2, MYC3, and MYC4) of *A. thaliana*. AIB is an abscisic acid (ABA)-inducible transcriptional repressor that negatively regulates JA signaling (Nakata et al., 2013). These domain features implicate that BrMYC2/3/4 belongs to the bHLH superfamily of TFs mediating the positive and negative regulation of plant development and various secondary metabolites synthesis.

### Evolutionary Analyses of BrMYC2/3/4

**Figure 2:**
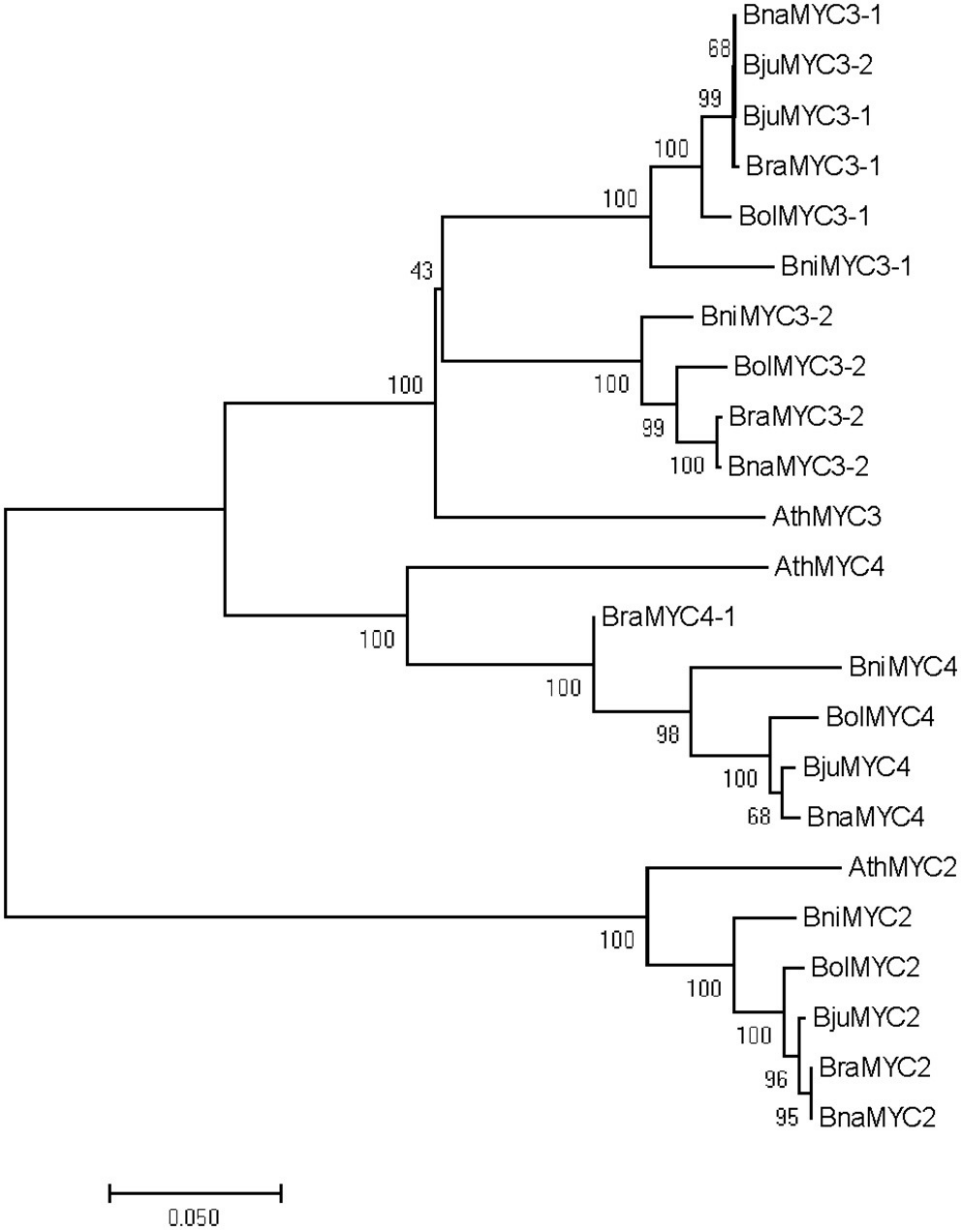
Phylogenetic tree of BrMYC2/3/4. Evolutionary analyses of full-length MYC2/3/4 coding sequences involved in the U's triangle of *Brassica* species. Numbers on the branches indicate the percentage of bootstrap support from 1,000 replicates; branch length represents the divergence distance. Ath, *Arabidopsis thalinala*; Bra, *Brassica rapa*; Bni, *Brassica nigra*; Bol, *Brassica oleracea*; Bna, *Brassica napus*; Bju, *Brassica juncea*.

A cladogram of MYC2/3/4 in the U's triangle species genomes was constructed according to the neighbor-joining method. As shown in Figure 2, *Brassicaceae* MYC2/3/4 family members exhibited a divergent evolution. BraMYC2 (*B. rapa*) forms a clade with BnaMYC2 (*B. napus*), and they form a clade with AthMYC2 (*A. thaliana*) by a high nodal support value. BraMYC3-1 forms a clade with *BnaMYC3-1, BjuMYC3-2* (*B. juncea*), and BjuMYC3-1. BraMYC3-2 forms a clade with BnaMYC3-2. BraMYC3-1 and BraMYC3-2 make up a clade with AthMYC3 by a high nodal support value. BraMYC4-1, 4-2, and 4-3 share an evolutionary lineage with the middle part, the first half, and a small part of the second half of AthMYC4, respectively. All these phylogenetic relationships are well in accordance with the classification based on morphological and biochemical characteristics of *Brassicaceae* plants.

### Transgenic Plants Obtain and Differential Expression of *BrMYC2/3/4* Genes

**Figure 3:**
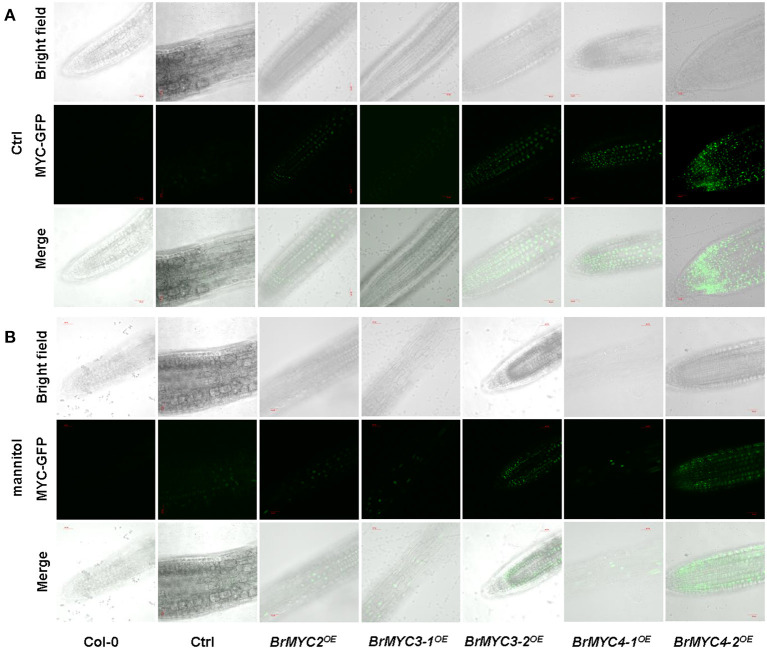
Green fluorescent protein (GFP) in the root tip of transgenic *Arabidopsis*, BrMYC2/3/4-GFP localizes to the nucleus. **(A,B)** Confocal images show the presence of BrMYC2/3/4-GFP fluorescence at the edges of root cells of 5-day-old transgenic *Arabidopsis* seedlings without **(A)** or with **(B)** plasmolysis induced by 1 M mannitol treatment for 5 min. Top panels, bright-field images; middle panels, fluorescence images of MYC-GFP; bottom panels, merged bright-field and fluorescence images. Bar = 30 μm.

To determine the obtain of transgenic plants, the root tip of resistant *Arabidopsis* was observed using spinning disk confocal microscopy. BrMYC2/3/4-GFP fusion protein fluorescence signal was observed under the control of CaMV 35S promoter (Figure 3).

Semi-quantitative RT-PCR was used to estimate the expression levels of five *BrMYC2/3/4* homologous genes in *BrMYC*^*OE*^ transgenic lines relative to Ctrl. Primers were designed from the gene-specific region (Supplementary Table 1). In the randomly selected three *BrMYC2/3/4*^*OE*^ transgenic lines, *BrMYC2/3/4* expression levels were all elevated (Supplementary Table 2). In *BrMYC2*^*OE*^*, BrMYC2* expression levels elevated from 8.61 to 11.07 folds relative to Ctrl. In *BrMYC3*^*OE*^, *BrMYC3-1* expression levels elevated from 5.72 to 7.50 folds, and *BrMYC3-2* expression levels elevated from 2.97 to 4.43 folds. In *BrMYC4*^*OE*^, *BrMYC4-1* expression levels elevated from 9.32 to 10.49 folds, and *BrMYC4-2* expression levels elevated from 11.32 to 11.43 folds.

### Effects of *BrMYC2/3/4* Overexpression on Seed Production

**Figure 4:**
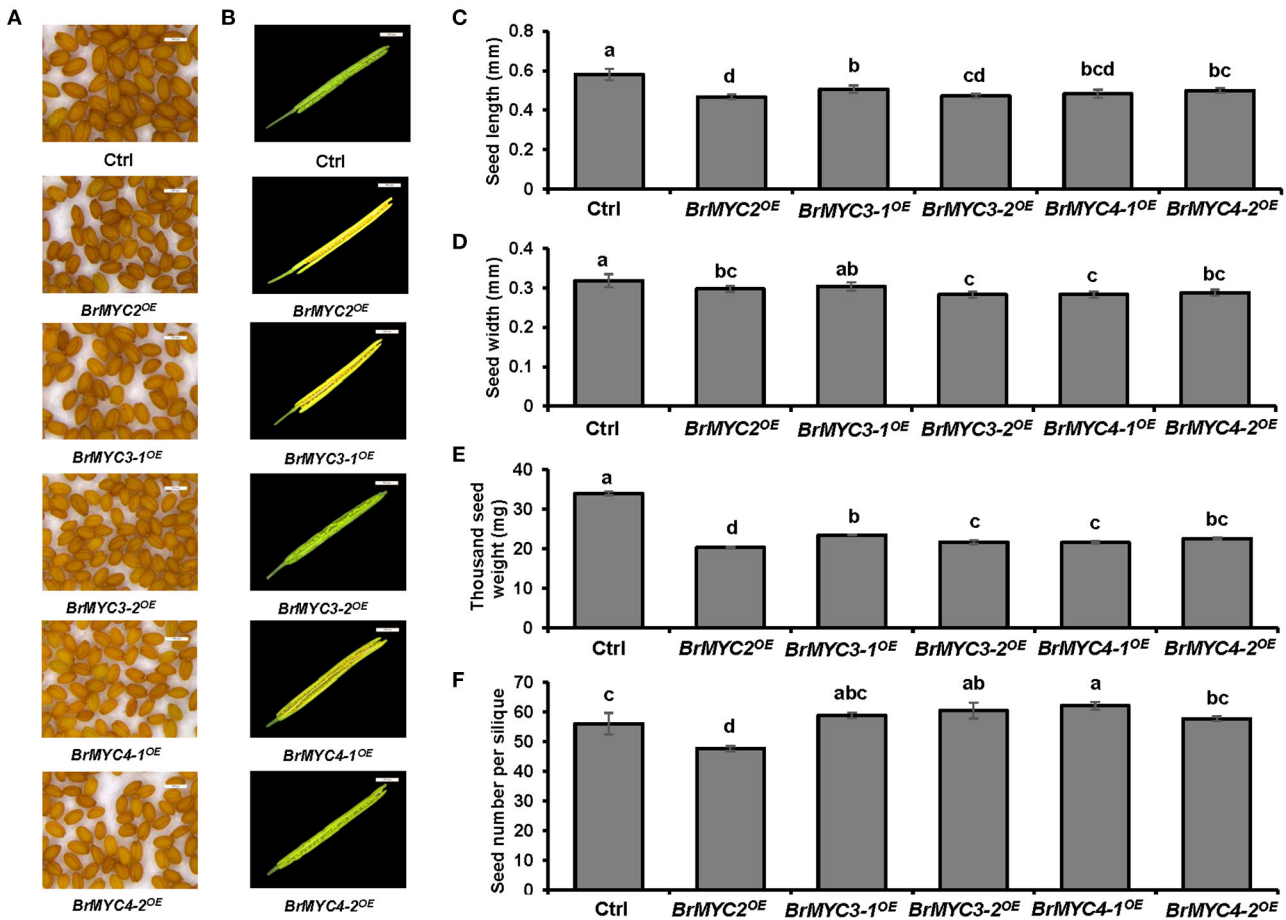
Seed production of T3 *Arabidopsis* lines transgenic for *BrMYC2/3/4* genes. **(A)** Phenotype of the mature seed. **(B)** Phenotype of decapsulated mature siliques. **(C)** Comparison of the seed length. **(D)** Comparison of the seed width. **(E)** Comparison of 1,000 seed weight. **(F)** Comparison of seed number per silique. Tested transgenic lines are Ctrl, *BrMYC2*^*OE*^, *BrMYC3-1*^*OE*^, *BrMYC3-2*^*OE*^, *BrMYC4-1*^*OE*^, and *BrMYC4-2*^*OE*^. Seeds and siliques were randomly selected and pictures were taken under the same conditions. Data are shown as mean ± SD from three independent biological replicates. ANOVA was used for statistical analysis, followed by Tukey's multiple comparison test (*p* < 0.05). Bar = 500 μm.

Since BrMYC4-3 was only 63 bp and its predicted domain function was completely different from others, only *BrMYC2/3-1/3-2/4-1/4-2* were cloned and transformed for further expression analysis. Transgenic *Arabidopsis* plants of Ctrl and *BrMYC2/3-1/3-2/4-1/4-2*^*OE*^ were analyzed for seed yield in terms of seed size (length and width), 1,000 seed weight, and seed number per silique. In order to minimize the effects of environmental factors on seed development, all plants were kept under identical growing conditions, such as temperature, light, water, and nutrition. As shown in Figure 4, there was a significant difference in seed production among different transgenic plants. *BrMYC2*^*OE*^ plant had the smallest seed length; there was no significant difference in seed length among *BrMYC3-2*^*OE*^, *BrMYC4-1*^*OE*^, and *BrMYC4-2*^*OE*^, all of which were significantly smaller than that of Ctrl (Figure 4C). *BrMYC3-2*^*OE*^ and *BrMYC4-1*^*OE*^ plants possessed the smallest seed width; there was no significant difference in seed width between *BrMYC2*^*OE*^ and *BrMYC4-2*^*OE*^, as well as between *BrMYC3-1*^*OE*^ and Ctrl (Figure 4D). Thousand seed weight significantly decreased in all the transgenic lines as compared with Ctrl (Figure 4E). *BrMYC2*^*OE*^ line contained the smallest seed weight, followed by *BrMYC3-2*^*OE*^, *BrMYC4-1*^*OE*^, *BrMYC4-2*^*OE*^, and *BrMYC3-1*^*OE*^. Thousand seed weight of *BrMYC3-1*^*OE*^ increased more than those of *BrMYC3-2*^*OE*^ and *BrMYC4-1*^*OE*^, while there was no significant difference among those of *BrMYC3-2*^*OE*^, *BrMYC4-1*^*OE*^, and *BrMYC4-2*^*OE*^, as well as between *BrMYC4-2*^*OE*^ and *BrMYC3-1*^*OE*^*. BrMYC2*^*OE*^ line contained the lowest seed number per silique, while all other transgenic lines had significantly higher seed numbers than Ctrl (Figures 4B,F). *BrMYC4-1*^*OE*^ line produced the highest seed number per silique followed by *BrMYC3-2*^*OE*^, *BrMYC3-1*^*OE*^, and *BrMYC4-2*^*OE*^. Taken the results together, constitutive expression of BrMYC2/3/4 TFs differentially affected the seed production with respect to the size, weight, and number of seed. Overexpression of *BrMYC3-1/3-2/4-1/4-2* had a negative effect on both the seed length and seed weight and a positive effect on seed number per silique. In contrast, overexpression of *BrMYC2* led to a negative effect on the seed length, seed weight, and seed number per silique.

### Effects of *BrMYC2/3/4* Overexpression on Plant Development

To determine if *BrMYC2/3/4* expression influences vegetative and reproductive development, we examined the phenotypes of transgenic *BrMYC2/3/4* plants at the seedling and bolting stage.

**Figure 5:**
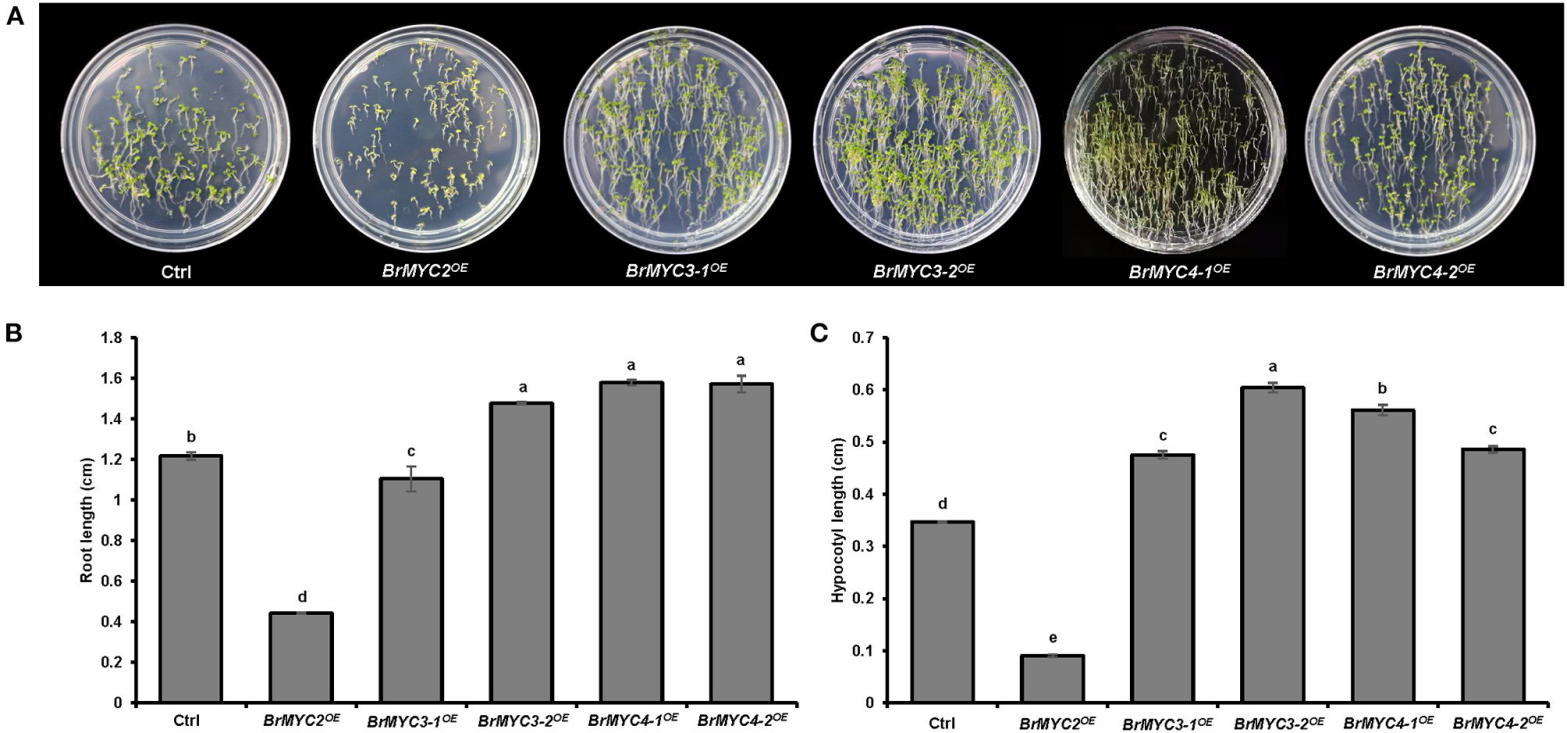
Root and hypocotyl length of T3 *Arabidopsis* lines transgenic for *BrMYC2/3/4* genes. **(A)** Phenotype of 6-day old seedlings. **(B)** Comparison of the root length. **(C)** Comparison of the hypocotyl length. Transgenic lines are Ctrl, *BrMYC2*^*OE*^, *BrMYC3-1*^*OE*^, *BrMYC3-2*^*OE*^, *BrMYC4-1*^*OE*^, and *BrMYC4-2*^*OE*^. Data are shown as mean ± SD from three independent biological replicates. ANOVA was used for statistical analysis, followed by Tukey's multiple comparison test (*p* < 0.05). Bar = 1 cm.

As for the vegetative stage, we studied the length of root and hypocotyl of seedlings. Significant differences in root and hypocotyl length were noted between Ctrl and *BrMYC2/3-1/3-2/4-1/4-2*^*OE*^ lines (Figure 5). *BrMYC2*^*OE*^ line exhibited the shortest root length, followed by *BrMYC3-1*^*OE*^, *BrMYC3-2*^*OE*^, *BrMYC4-1*^*OE*^, and *BrMYC4-2*^*OE*^ (Figure 5B). Root length of *BrMYC2*^*OE*^ and *BrMYC3-1*^*OE*^ lines was shorter than that of Ctrl. Although there was no significant difference among *BrMYC3-2*^*OE*^, *BrMYC4-1*^*OE*^, and *BrMYC4-2*^*OE*^ lines, their root length was significantly longer than that of Ctrl. *BrMYC2*^*OE*^ had also the shortest length of hypocotyl, followed by *BrMYC3-1*^*OE*^, *BrMYC4-2*^*OE*^, *BrMYC4-1*^*OE*^, and *BrMYC3-2*^*OE*^ (Figure 5C). *BrMYC2*^*OE*^ line showed a shorter hypocotyl length than Ctrl, whereas transgenic *BrMYC3-1*^*OE*^, *BrMYC3-2*^*OE*^, *BrMYC4-1*^*OE*^, and *BrMYC4-2*^*OE*^ lines exhibited longer hypocotyl lengths than Ctrl. Thus, overexpression of *BrMYC2*/*3-1* and *BrMYC3-2/4-1/4-2* appears to inhibit and promote root elongation in *Arabidopsis*, respectively. Overexpression of *BrMYC2* and *BrMYC3-1/3-2/4-1/4-2* seems to inhibit and foster hypocotyl elongation of *Arabidopsis*, respectively.

**Figure 6:**
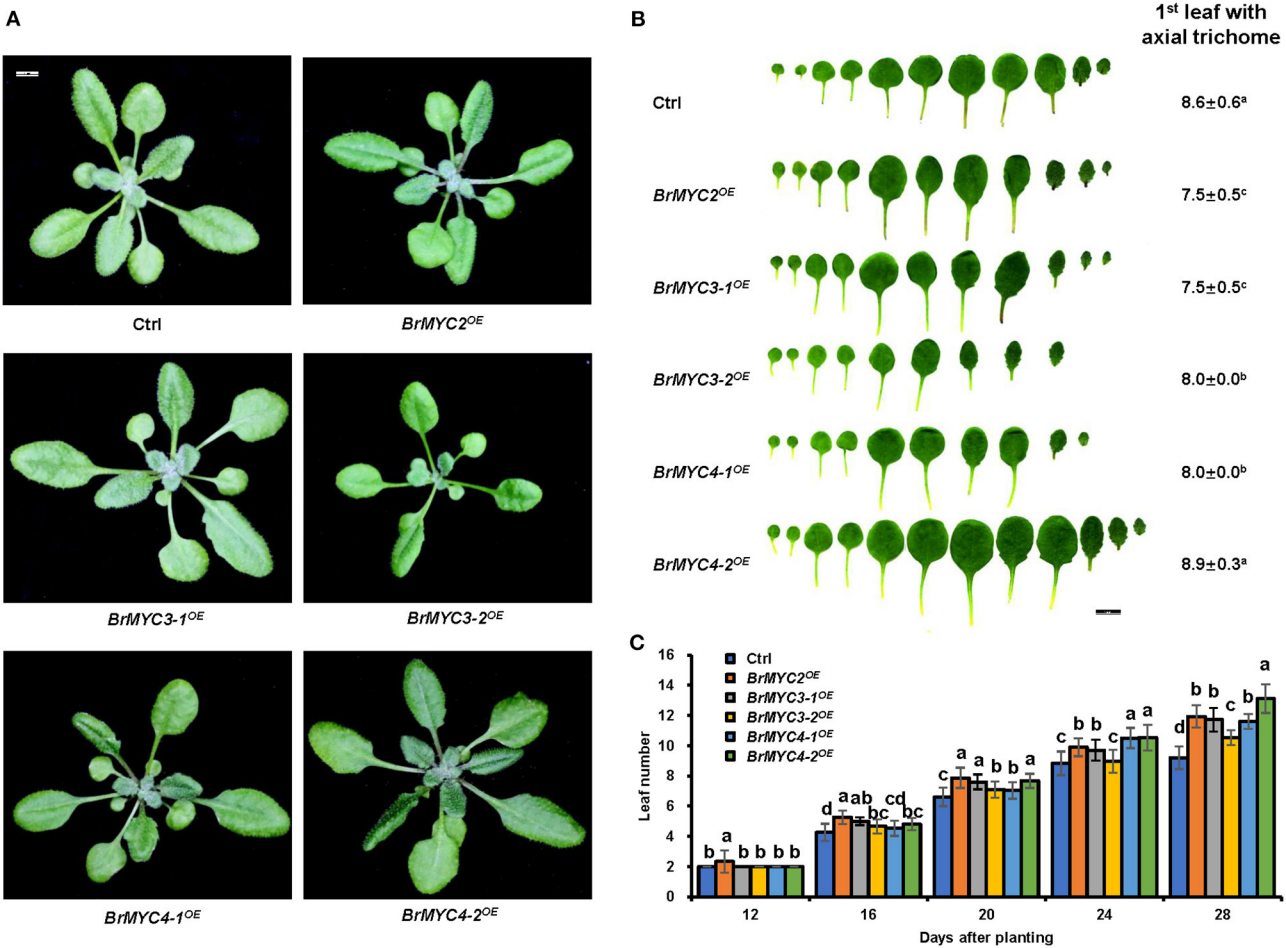
The effect of *BrMYC2/3/4* expression on the growth of *Arabidopsis*. **(A)** Phenotype of 28-day old transgenic *Arabidopsis*. **(B)** Phenotype of leaf shape and abaxial trichome of fully expanded rosette leaves. The average numbers of the first leaf showing abaxial trichomes are presented. Different superscript letters on the numbers denote a significant difference between the transgenic lines. **(C)** Leaf initiation rate of transgenic *Arabidopsis* in short days. Leaf numbers were scored at 12, 16, 20, 24, and 28 days after planting. Transgenic lines are Ctrl, *BrMYC2*^*OE*^, *BrMYC3-1*^*OE*^, *BrMYC3-2*^*OE*^, *BrMYC4-1*^*OE*^, and *BrMYC4-2*^*OE*^. Data are shown as mean ± SD from three independent biological replicates. ANOVA was used for statistical analysis, followed by Tukey's multiple comparison test (*p* < 0.05). Bar = 1 cm.

About 3–4 weeks after seeds were sown, *Arabidopsis* seedlings transitioned from juvenile to adult vegetative growth phase (Figure 6). The leaf shape of transgenic plants was normal and indistinguishable from that of Ctrl. *BrMYC2/3-1/3-2/4-1*^*OE*^ lines produced smaller number of the first leaves having trichomes than Ctrl, whereas *BrMYC4-2*^*OE*^ produced a larger number of them on leaf than Ctrl (Figure 6B). For the leaf initiation rate, *BrMYC2/3-1/4-1/4-2*^*OE*^ was significantly faster than Ctrl, but *BrMYC3-2*^*OE*^ was similar to Ctrl (Figure 6C).

**Figure 7:**
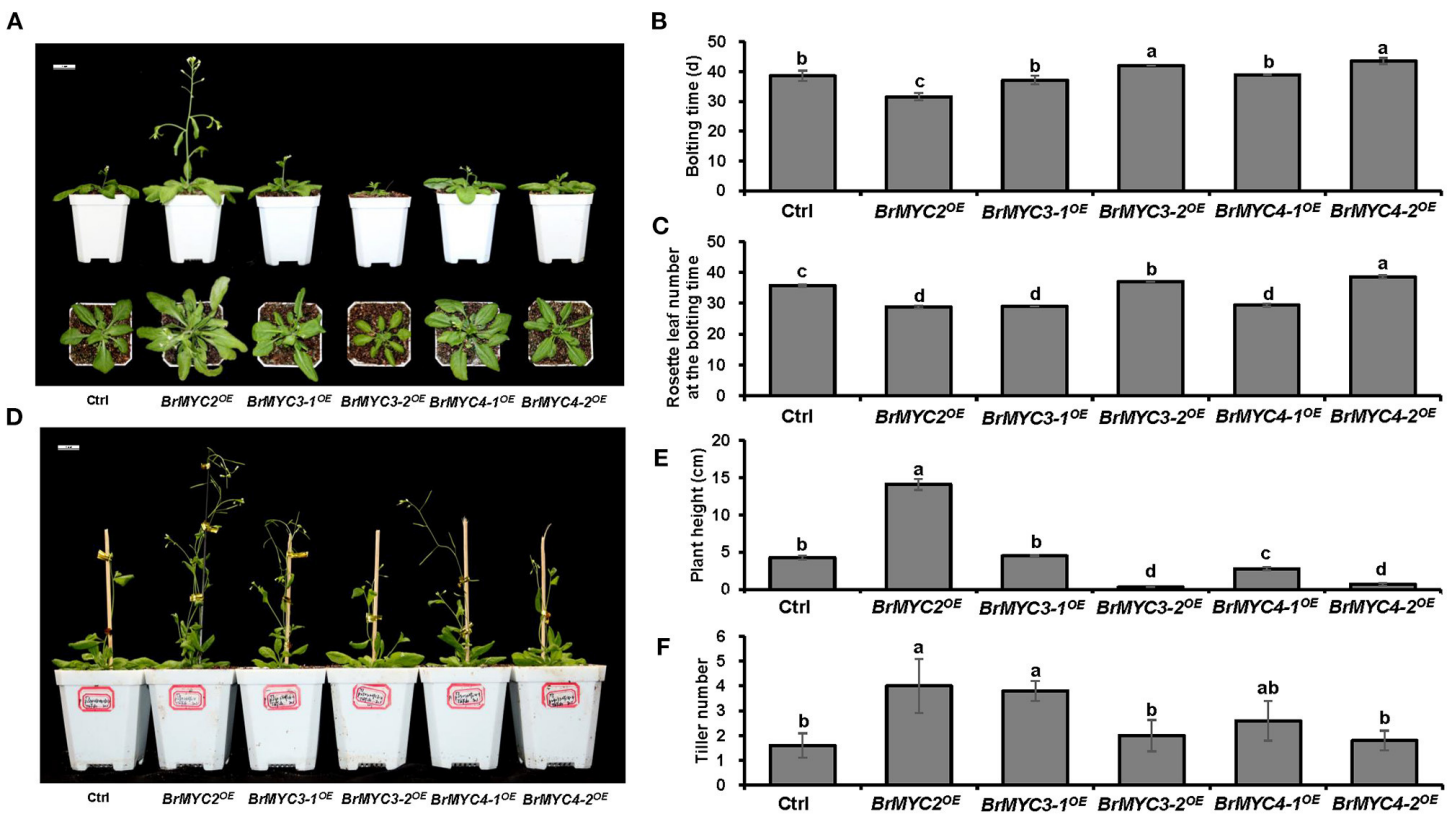
Plant height and flowering time of transgenic *Arabidopsis*. **(A)** Phenotype of 3-week-old *Arabidopsis*. **(B)** Bolting time. **(C)** Rosette leaf number at the bolting time. **(D)** Phenotype of 7-week-old *Arabidopsis*. **(E)** Plant height. **(F)** Tiller number. Transgenic lines are Ctrl, *BrMYC2*^*OE*^, *BrMYC3-1*^*OE*^, *BrMYC3-2*^*OE*^, *BrMYC4-1*^*OE*^, and *BrMYC4-2*^*OE*^. Data are shown as mean ± SD from three independent biological replicates. ANOVA was used for statistical analysis, followed by Tukey's multiple comparison test (*p* < 0.05). Bar = 1 cm.

As for the reproductive stage, we studied bolting time, rosette leaf number at the bolting time, plant height, and tiller number (Figure 7). *BrMYC2*^*OE*^ line had significantly faster bolting time, while *BrMYC3-2*^*OE*^ and *BrMYC4-2*^*OE*^ lines had significantly slower bolting time than Ctrl. Both *BrMYC3-1*^*OE*^ and *BrMYC4-1*^*OE*^ were similar to Ctrl in bolting time (Figures 7A,B). The number of rosette leaves at bolting time significantly decreased in *BrMYC2/3-1/4-1*^*OE*^ lines but increased in *BrMYC3-2/4-2*^*OE*^ lines as compared with Ctrl (Figures 7A,C). Previously, flowering time was recorded as the number of rosette leaves at bolting, with the observation of late flowering plants having more rosette leaves (Lopez-Vernaza et al., 2012). Thus, it appears that *BrMYC2*^*OE*^, *BrMYC3-1*^*OE*^, and *BrMYC4-1*^*OE*^ promoted flowering time, while *BrMYC3-2*^*OE*^ and *BrMYC4-2*^*OE*^ inhibited it in *Arabidopsis*. For the plant height, *BrMYC2*^*OE*^ significantly increased, while *BrMYC3-2*^*OE*^, *BrMYC4-1*^*OE*^, and *BrMYC4-2*^*OE*^ significantly decreased as compared with Ctrl. And there was no significant difference between *BrMYC3-2*^*OE*^ and *BrMYC4-2*^*OE*^, as well as between *BrMYC3-1*^*OE*^ and Ctrl (Figures 7A,E). Tiller number of *BrMYC2*^*OE*^ and *BrMYC3-1*^*OE*^ was significantly higher than Ctrl and no significant difference was noted among *BrMYC3-2*^*OE*^, *BrMYC4-1*^*OE*^, *BrMYC4-2*^*OE*^, and Ctrl (Figures 7A,F). Thus, expression of *BrMYC2* only promoted the growth of both shoot elongation and plant height after fluorescence with faster bolting time.

### Analysis of GSs Contents

In order to investigate the effect of *BrMYC2/3/4* transgene expression on GSs metabolism, GSs profiles in the leaves of T3 lines were determined using HPLC. Transgenic lines exhibited significantly increased levels of most of the short- and long-chain AGSs and IGSs when compared to those of Ctrl (Tables 1, 2). Glucoiberin (GBR), derived from homomethionine, increased by 2.5-fold in *BrMYC2*^*OE*^ line. AGS derived from dihomomethionine, such as glucoerucin (GEC) and glucoraphanin (GRN), increased by 3.1- and 2.2-fold in *BrMYC2*^*OE*^ line, respectively. Glucoalyssin (GAS), derived from trihomomethionine, increased by 2.2-fold in *BrMYC2*^*OE*^ line. Glucohirsutin (GHT), derived from pentahomomethionine, increased by 3.9-fold in *BrMYC2*^*OE*^, 1.9-fold in *BrMYC3-1*^*OE*^, and 1.8-fold in *BrMYC4-1*^*OE*^ lines. The major IGS such as glucobrassicin (GBC), 4MeGBC, and neoglucobrassicin (NeoGBC) increased by 1.6-, 8.9- and 1.2-fold in *BrMYC2*^*OE*^ line, respectively. 4MeGBC increased by 2.9-fold in *BrMYC3-1*^*OE*^ line. NeoGBC increased by 4.7-, 1.9-, 3.3-, and 1.9-fold in *BrMYC3-1/3-2/4-1/4-2*^*OE*^ lines, respectively. As shown in Table 3, AGS increased by 2.9-, 1.7-, and 1.6-fold in *BrMYC2/3-1/4-1*^*OE*^ lines, respectively. IGS increased by 4.5- and 2.9-fold in *BrMYC2/3-1*^*OE*^ lines, respectively. Total GS increased by 3.0-, 1.7-, and 1.6-fold in *BrMYC2/3-1/4-1*^*OE*^ lines, respectively. Hence, levels of most AGS and IGS significantly increased in all the lines except for *BrMYC4-2*^*OE*^.

**Table 1:** Individual aliphatic glucosimolate (GS) content (μmol·g^−1^ DW).

	**GBR**	**GRN**	**GAS**	**GNA**	**GBV**	**GEC**	**GHT**
Ctrl	0.36 ± 0.03^b^	0.39 ± 0.02^b^	2.72 ± 0.15^c^	0.15 ± 0.01^a^	0.10 ± 0.03^a^	0.29 ± 0.02^b^	2.92 ± 0.21^c^
*BrMYC2^*OE*^*	0.90 ± 0.29^a^	0.83 ± 0.20^a^	6.05 ± 0.09^a^	0.27 ± 0.13^a^	0.12 ± 0.03^a^	0.88 ± 0.26^a^	11.32 ± 0.11^a^
*BrMYC3-1^*OE*^*	0.56 ± 0.15^ab^	0.67 ± 0.07^ab^	3.95 ± 0.05^b^	0.43 ± 0.06^a^	0.15 ± 0.01^a^	0.65 ± 0.15^ab^	5.51 ± 0.28^b^
*BrMYC3-2^*OE*^*	0.57 ± 0.06^ab^	0.47 ± 0.02^ab^	3.80 ± 0.40^b^	0.23 ± 0.01^a^	0.11 ± 0.01^a^	0.22 ± 0.08^b^	3.61 ± 0.28^c^
*BrMYC4-1^*OE*^*	0.50 ± 0.07^ab^	0.49 ± 0.24^ab^	3.52 ± 0.32^bc^	0.39 ± 0.18^a^	0.12 ± 0.06^a^	0.60 ± 0.26^ab^	5.28 ± 0.30^b^
*BrMYC4-2^*OE*^*	0.46 ± 0.17^ab^	0.45 ± 0.03^ab^	3.23 ± 0.36^bc^	0.20 ± 0.02^a^	0.09 ± 0.03^a^	0.16 ± 0.06^b^	3.16 ± 0.41^c^

*Values represent the means of triplicate samples ± standard deviation. ANOVA was used for statistical analysis, followed by Tukey's multiple comparison test. Significant differences of GS levels among different samples are labeled with different superscript lower-case letters (p < 0.05)*.

*GAS, glucoalyssin; GBR, glucoiberin; GBV, glucoiberverin; GEC, glucoerucin; GHT, glucohirsutin; GNA, gluconapin; GRN, glucoraphanin*.

**Table 2:** Individual indole GS (IGS) content (μmol·g^−1^ DW).

	**4-OHGBC**	**GBC**	**4MeGBC**	**NeoGBC**
Ctrl	0.03 ± 0.00^ab^	0.04 ± 0.00^b^	0.11 ± 0.01^c^	0.12 ± 0.01^d^
*BrMYC2^*OE*^*	0.09 ± 0.02^a^	0.06 ± 0.01^a^	0.96 ± 0.20^a^	0.36 ± 0.00^a^
*BrMYC3-1^*OE*^*	0.05 ± 0.02^ab^	0.05 ± 0.01^ab^	0.51 ± 0.12^b^	0.34 ± 0.02^ab^
*BrMYC3-2^*OE*^*	0.05 ± 0.01^b^	0.03 ± 0.01^b^	0.20 ± 0.06^bc^	0.21 ± 0.01^c^
*BrMYC4-1^*OE*^*	0.03 ± 0.01^b^	0.03 ± 0.00^b^	0.36 ± 0.05^bc^	0.31 ± 0.01^b^
*BrMYC4-2^*OE*^*	0.04 ± 0.01^b^	0.03 ± 0.00^b^	0.21 ± 0.01^bc^	0.24 ± 0.01^c^

*Values represent the means of triplicate samples ± standard deviation. ANOVA was used for statistical analysis, followed by Tukey's multiple comparison test. Significant differences of GS levels among different samples are labeled with different superscript lower-case letters (p < 0.05)*.

*4MeGBC, 4-methoxyglucobrassicin; 4-OHGBC, 4-hydroxyglucobrassicin; GBC, glucobrassicin; NeoGBC, neoglucobrassicin*.

**Table 3:** Total glucosimolate (GS) content (μmol·g^−1^ DW).

	**AGS**	**IGS**	**Total GS**
Ctrl	6.94 ± 0.47^d^	0.32 ± 0.04^c^	7.26 ± 0.50^d^
*BrMYC2^*OE*^*	20.37 ± 0.83^a^	1.46 ± 016^a^	21.82 ± 0.99^a^
*BrMYC3-1^*OE*^*	11.52 ± 0.87^b^	0.94 ± 0.09^b^	12.46 ± 0.96^b^
*BrMYC3-2^*OE*^*	9.01 ± 0.87^cd^	0.49 ± 0.09^c^	9.50 ± 0.96^cd^
*BrMYC4-1^*OE*^*	10.91 ± 1.41^ab^	0.73 ± 0.08^bc^	11.63 ± 1.49^bc^
*BrMYC4-2^*OE*^*	7.76 ± 1.09^d^	0.51 ± 0.04^c^	8.27 ± 1.12^d^

*Values represent the means of triplicate samples ± standard deviation. ANOVA was used for statistical analysis, followed by Tukey's multiple comparison test. Significant differences of GS levels among different samples are labeled with different superscript lower-case letters (p < 0.05)*.

*AGS, aliphatic glucosinolate; IGS, indole glucosinolate*.

### Anti-fungal Activity

**Figure 8:**
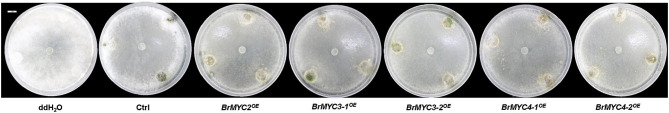
Inhibition of *S. sclerotiorum* growth by the lyophilized powder of each transgenic *A. thaliana* line on PDA. From left to right are: ddH_2_O, Ctrl, *BrMYC2*^*OE*^, *BrMYC3-1*^*OE*^, *BrMYC3-2*^*OE*^, *BrMYC4-1*^*OE*^, and *BrMYC4-2*^*OE*^. One sclerotium of *S. sclerotiorum* was placed in the center of each dish. Fungal hyphae growth was visible as white cottony mycelium plaque. Bar = 1 cm.

To study the extent of resistance of transgenic lines to *S. sclerotiorum*, 25 mg of the lyophilized powder of each transgenic line's leaf tissue was uniformly sprinkled around the hyphae. The visible growth of cottony mycelium of plaque was observed after 72 h of *S. sclerotiorum* incubation (Figure 8). The thinnest plaque was noted in *BrMYC2*^*OE*^ line, followed by *BrMYC3-1*^*OE*^, *BrMYC4-1*^*OE*^, *BrMYC3-2*^*OE*^, and *BrMYC4-2*^*OE*^. Ctrl displayed much thinner plaque than water control, suggesting that endogenous basal levels of GSs present in the vector control line inhibited the growth of *S. sclerotiorum* to a certain degree. However, Ctrl showed thicker plaques than *BrMYC2/3/4*^*OE*^ lines. This may result from the suppression of the mycelium growth by higher contents of GSs accumulated in *BrMYC2/3/4*^*OE*^ lines.

## Discussion

Our *in-silico* analysis indicated that BrMYC2/3-1/3-2/4-2/4-3 are unstable hydrophobic and acidic proteins, whereas BrMYC4-1 is an unstable hydrophobic and acidic basic protein. BrMYC2/3/4 TFs were predicted to contain the bHLH AtAIB-like domain whose protein is involved in the positive regulation of ABA signaling in *Arabidopsis* (Figure 1). Notably, the molecular weight of BrMYC4-3 was far smaller and its domain function was very different from others. Phylogenetic relationship analyses indicated that BrMYC2/3/4 were closely related to AthMYC2/3/4 orthologs from the *Brassicaceae* family.

**Figure 9:**
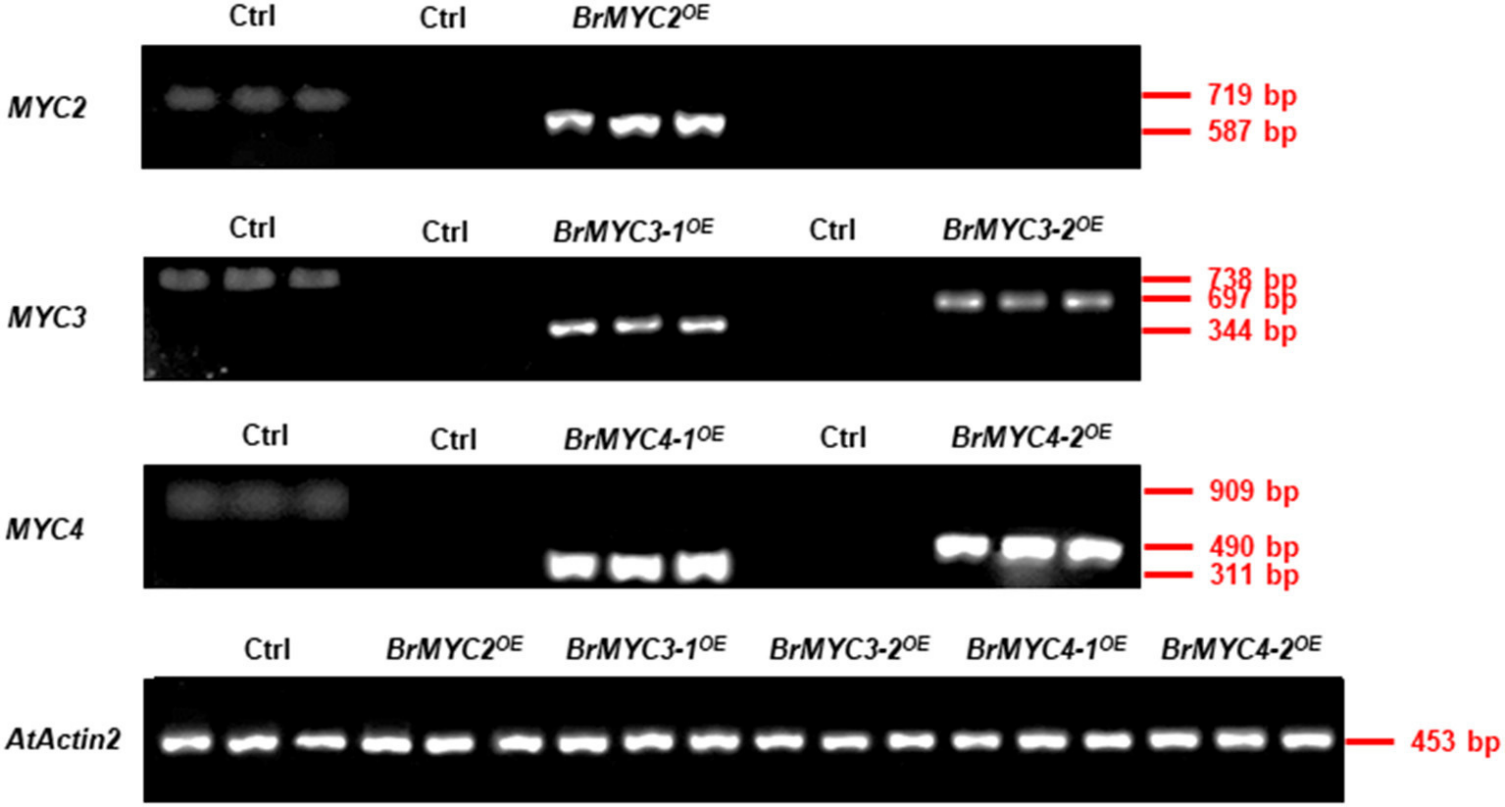
Semi-quantitative RT-PCR analysis of *BrMYC2/3/4* genes in 8-day-old transgenic *Arabidopsis*. The left column of Ctrl with three lanes used primers from corresponding *Arabidopsis* genes, and the other's Ctrl used primers from corresponding *Brassica rapa* genes. *AtActin2* serves as an internal control.

Transgenic *Arabidopsis* plants of BrMYC2/3/4 were verified by observation of GFP fluorescence (Figure 3). Expression levels of five *BrMYC2/3/4* homologous genes, all elevated relative to Ctrl (Figure 9).

When expressed in *Arabidopsis* under the control of 35S promoter, each member of the *BrMYC2/3-1/3-2/4-1/4-2* transgenes differentially influenced root and shoot elongation, vegetative phase change, flowering time, plant height and tiller number after fluorescence, and seed production. Seed size is a key agronomic trait that determines the grain yield and breeding of plants (Shirley et al., 2019). The number and weight of the seed ultimately determine seed yield (Jiang et al., 2020). In this study, we found that as a result of overexpression of each of *BrMYC2/3-1/3-2/4-1/4-2*, both the seed length and seed weight decreased (Figures 4C,E), while seed number per silique increased except for *BrMYC2*^*OE*^ as compared to Ctrl (Figure 4F). The results appear to be consistent with a finding that *MYC2, MYC3*, and *MYC4* act additively during seed development and that triple mutants produced the largest seeds with more seed storage proteins, while the seeds of single and double mutants were much larger than those of wild type (Gao et al., 2016). Hence, overexpression of the individual *BrMYC2/3-1/3-2/4-1/4-2* family genes appears to have a negative effect on seed size and seed weight.

To examine the effect of *BrMYC* expression on plant development, the lengths of root and hypocotyl of seedlings were measured. We found that *BrMYC2* expression significantly inhibited both root and hypocotyl elongation, whereas *BrMYC3-2/4-1/4-2* expression significantly promoted both root and hypocotyl elongation when compared to Ctrl (Figure 5). In contrast, *BrMYC3-1* expression significantly inhibited root elongation but promoted hypocotyl elongation. Thus, expression of the duplicated copies of *BrMYC3, BrMYC3-1*, and *BrMYC3-2*, did not lead to the same effect on the root length but on the hypocotyl length. This is an interesting observation, suggesting that they have divergent functions and regulatory roles in plant development. In *A. thaliana*, the transition from juvenile to adult vegetative phase referred to as “vegetative phase change” is signified by the formation of trichomes on the abaxial side of leaf blade and an increase in the leaf length/width ratio during shoot development (Xu et al., 2019). Our studies on abaxial trichome production and leaf initiation rate as shown in Figure 6 suggested that *BrMYC2/3-1/4-1*^*OE*^ positively regulate the time of vegetative phase change in *Arabidopsis*, since the less number of the first-formed leaves having abaxial trichomes relative to control was correlated with promoting floral transition (Chien and Sussex, 1996). Previous studies showed that the plant height determines its ability to compete for light and therefore often correlates with the leaf mass and seed production, and that stem growth is initiated once the plant becomes reproductive and continues until termination of the inflorescence meristems (Serrano-Mislata et al., 2017; Moles et al., 2019). Based on the bolting time and rosette leaf number at bolting time, it appears that *BrMYC2*/*3-1*/*4-1*^*OE*^ promoted flowering time, while *BrMYC3-2*/*4-2*^*OE*^ inhibited it (Figure 7). Hence, expression of each set of the duplicated copies of *BrMYC3 (3-1/3-2)* and *BrMYC4 (4-1/4-2)* led to the opposite action on flowering time, suggesting that they have divergent function and regulatory roles in fluorescence. After flowering, *BrMYC2*^*OE*^ promoted the growth of the plant height, whereas *BrMYC3-2/4-1/4-2*^*OE*^ repressed it, suggesting that the three MYC TFs play different roles in signaling pathways. Notably, *BrMYC2/3-1*^*OE*^ displayed a remarkable increase in tiller number after fluorescence. The earlier flowering phenotype of overexpressed *BrMYC2* seems to be consistent with a previous finding that expression of all three *MYC2/3/4* was required to inhibit flowering in *Arabidopsis* (Wang et al., 2017). Along this line, it was reported that MYC2 regulates diverse functions within the JA signaling pathway and that MYC2 forms homo- and/or heterodimers with MYC3 and MYC4 and binds to the conserved G-box present in the promoters of JA-responsive genes (Kazan and Manners, 2013). Moreover, Zhai et al. (2013) reported that high and low accumulation of the MYC2 protein correlated with positive regulation of early wound-responsive genes and negative regulation of late pathogen-responsive genes, respectively, and that MYC activity was further regulated by phosphorylation and the ubiquitin-proteasome system-mediated proteolysis. Accordingly, a wide range of differential effects of each *BrMYC2/3/4* expression on the root and shoot elongation, vegetative phase change, flowering time, and seed production implicates MYC-mediated complex signaling networks for positive and negative regulation of plant growth and development to connect environmental stresses with developmental signals at both transcriptional and post-transcription levels.

It was reported that bHLH-type MYC TFs, participating in crosstalk among JA and other hormones as well as environmental signals, allow the coordinated and rapid regulation of GS genes (Mitreiter and Gigolashvili, 2021). MYC-interaction motif mediates interactions between MYC and MYB TFs in *A. thaliana* that are critical for constitutive and induced GS biosynthesis (Millard et al., 2021). MYC3/4 act additively with MYC2 to stimulate GS biosynthesis (Guo et al., 2013). In *Arabidopsis*, MYC2 binds directly to the promoters of several GS biosynthesis genes, and that MYC2/3/4 interact directly with GS-related MYBs (Schweizer et al., 2013). Therefore, MYC2/3/4 are involved in the regulation of GS biosynthesis. In line with these findings, our study showed that transgenic *Arabidopsis* plants overexpressing *BrMYC2/3-13-2//4-1* accumulated significantly higher levels of GS relative to Ctrl (Tables 1–3). Levels of AGS remarkably increased in *BrMYC2/3-1/4-1*^*OE*^ lines. GHT content also significantly rose in *BrMYC3-1*^*OE*^ and *BrMYC4-1*^*OE*^ lines. Levels of IGS were strikingly elevated in *BrMYC2/3-1*^*OE*^ lines. NeoGBC content significantly increased in *BrMYC2/3-1/3-2/4-1/4-2*^*OE*^ lines. Overall, total GS considerably increased in *BrMYC2/3-1/3-2/4-1*^*OE*^ lines. *BrMYC2* was far more effective to positively regulate the biosynthesis of both AGS and IGC than *BrMYC3* and *BrMYC4*.

Modulation of GS content affected plant resistance to pathogenic infection in *Arabidopsis* (Madloo et al., 2019). *Brassicaceae* plants typically produce GSs which are hydrolyzed by myrosinases upon tissue damage to generate ITCs, toxic bioactive compounds acting against broad pathogens. 4MeGBC was activated by the atypical PEN2 myrosinase (a type of β-thioglucoside glucohydrolase) for antifungal defense (Bednarek et al., 2009). Fungal infection triggered the accumulation of both IGS and AGS in *B. rapa* (Abdel-Farid et al., 2010). In *B. rapa, S. sclerotiorum* inoculation *in vivo* induced GS accumulation and increased GS biosynthesis-related proteins (Teng et al., 2021). In our study, the lyophilized leaf powder of each *Arabidopsis* transgenic line was uniformly sprinkled around the hyphae in order to assess anti-fungal activity against *S. sclerotiorum* (Figure 8). PDA plate sprinkled with lyophilized powder of *BrMYC2*^*OE*^ line showed the thinnest fungal hyphae plaque, followed by those of *BrMYC3-1*^*OE*^ and *BrMYC4-1*^*OE*^ lines. Such a varying degree of fungal resistance exhibited by the overexpression lines seemed to be in line with the accumulation levels of GS. IGSs such as GBC, 4MeGBC, and NeoGBC, as well as GHT accumulated at a higher level in the order of *BrMYC2*^*OE*^, *BrMYC3-1*^*OE*^, and *BrMYC3-1*^*OE*^ lines (Table 2). GBC, 4MeGBC, and NeoGBC were not metabolized by *S. sclerotiorum* (Pedras and Hossain, 2011). Besides, a previous study showed that aliphatic ITCs inhibited the growth of *S. sclerotiorum in vitro*, and that anti-microbial activity of AGS-derived isothiocyanates was dependent on side chain elongation and modification, with glucohirsutin (GHT) being most toxic to *S. sclerotiorum* (Stotz et al., 2011).

## Conclusions

In summary, *Arabidopsis* plants expressing each of the five *MYC* paralogous genes of Chinese cabbage exhibited a wide range of differing phenotypes with respect to the root and shoot elongation, vegetative phase change, flowering time, plant height and tiller number right after flowering, and seed production. Despite the wide variation of phenotypes between the transgenic lines, all of the lines except for *BrMYC4-2*^*OE*^ exhibited shorter seed length, less seed weight, higher accumulation of GSs, and resistance to *S. sclerotiorum* than Ctrl. Notably, the highest GSs level accumulated in *BrMYC2*^*OE*^ line was correlated with the highest extent of resistance to the necrotic fungal pathogen *S. sclerotiorum*. Unlike *BrMYC3-1/3-2/4-/4-2, BrMYC2* expression stimulated the growth of plant height after fluorescence with a faster bolting time. The results presented here indicate that despite the complexity of GSs biosynthesis and metabolism regulated in the positive and negative manner by many other TFs, *BrMYC2* expression alone was far more effective to positively regulate the biosynthesis of both AGS and IGC than BrMYC3 and BrMYC4, and thus may provide the beneficial effects on the plant growth and development *via* resistance to fungal pathogens.

## Data Availability Statement

The original contributions presented in the study are included in the article/Supplementary Material, further inquiries can be directed to the corresponding author.

## Author Contributions

YZ conceived and designed the experiments and performed the analysis with discussions. ZT performed the experiment and data analysis. ZT, YY, and ZZ contributed to manuscript preparation. ZT and YZ wrote the original draft. S-BH, WZ, and ZZ edited the manuscript. All authors contributed to the article and approved the submitted version.

## Conflict of Interest

The authors declare that the research was conducted in the absence of any commercial or financial relationships that could be construed as a potential conflict of interest.

## Publisher's Note

All claims expressed in this article are solely those of the authors and do not necessarily represent those of their affiliated organizations, or those of the publisher, the editors and the reviewers. Any product that may be evaluated in this article, or claim that may be made by its manufacturer, is not guaranteed or endorsed by the publisher.

## Abbreviations

4MeGBC, 4-methoxyglucobrassicin: 4-methoxy-3-indolylmethyl-glucosinolate
4-OHGBC, 4-hydroxyglucobrassicin: 4-hydroxy-3-indolylmethyl-glucosinolate
ABA: abscisic acid
AGSs: aliphatic glucosinolates
AIB: ABA-inducible bHLH-TYPE
Ath: *Arabidopsis thaliana*
bHLH: basic helix–loop–helix
Bju: *Brassica juncea*
Bna: *Brassica napus*
Bni: *Brassica nigra*
Bol: *Brassica oleracea*
Bra: *Brassica rapa*
*BrMYC2/3/4*^*OE*^: transgenic *Arabidopsis* plants carrying *BrMYC2/3/4* genes
CDD: conserved domain database
cDNA: complementary DNA
COI1: coronatine insensitive1
Ctrl: transgenic *Arabidopsis* plants carrying an empty vector pCAMBIA2302
ddH_2_O: double-distilled water
GAS, glucoalyssin: 5-methylsulphinylpentyl-glucosinolate
GBC, glucobrassicin: 3-indolylmethyl-glucosinolate
GBR, glucoiberin: 3-methylsulphinylpropyl-glucosinolate
GBV, glucoiberverin: 3-methylthiopropyl-glucosinolate
GEC, glucoerucin: 4-methylthiobutyl-glucosinolate
GFP: green fluorescent protein
GHT, glucohirsutin: 8-methylsulphinyloctyl-glucosinolate
GNA, gluconapin: 3-butenyl-glucosinolate
GRN, glucoraphanin: 4-methylsulphinylbutyl-glucosinolate
GSs: glucosinolates
HLH: helix–loop–helix
I3C: indole-3-carbinol
IGSs: indole glucosinolates
ITCs: isothiocyanates
JA: jasmonic acid
Jas: jasmonates
JAZs: JAZIM-domains
MEGA7 software: molecular evolutionary genetics analysis software version 7. 0
MYB: v-myb avian myeloblastosis viral oncogene homolog
MYCs: myelocytomatosis proteins
NCBI: National Center for Biotechnology Information
NeoGBC, neoglucobrassicin: 1-methoxy-3-indolylmethyl-glucosinolate
OE: overexpression
PDA: potato dextrose agar
pI: isoelectric point
TFs: transcription factors.
