# Supplementary material for: Effects of BrMYC2/3/4 on Plant Development, Glucosinolate Metabolism, and Sclerotinia sclerotiorum Resistance in Transgenic Arabidopsis thaliana
Source: Front Plant Sci. 2021 Sep 3;12:707054. doi: 10.3389/fpls.2021.707054 (PMC8446384; doi:10.3389/fpls.2021.707054)
Supplement: Supplementary file 1 [file Data_Sheet_1.PDF]

Supplemental materials

Supplemental Table 1 Sequencing primers of *MYC2/3/4* and *Actin2* genes

Number	Primer name	Primer sequences (5' to 3')
1	AtMYC2-F	GCGATGGAGATTCCGGCACA
2	AtMYC2-R	GGTTCGTTAGATCCAGGTGT
3	AtMYC3-F	ATCTCTCTTTCCTCCACCAC
4	AtMYC3-R	GATTCGATTCCGGTGTTCGT
5	AtMYC4-F	GTCTCCGACGAATGTTCAAG
6	AtMYC4-R	GAAGTTGAGTCATTTCCACC
7	BrMYC2-F	CTCCACCGCCGTTTTCAACA
8	BrMYC2-R	GATGGTGCTTGAACTACCAC
9	BrMYC3-1-F	CTCTCTCCACCGGATGCTCA
10	BrMYC3-1-R	GATTCGAGCTCGAGCTCTGT
11	BrMYC3-2-F	AGGAGGAGGAGTCGGAGTCT
12	BrMYC3-2-R	ATCACTCTCGCCGGATTTCG
13	BrMYC4-1-F	TAGAATCGCGGCTGAACCG
14	BrMYC4-1-R	AACTTTTCACATTCCCATCC
15	BrMYC4-2-F	TTCAACTAACCGACCACCAC
16	BrMYC4-2-R	TCCCGCCTCGTCGGAAGA
17	AtActin2-F	AATTACCCGATGGGCA
18	AtActin2-R	TCATACTCGGCCTTGGA

Supplemental Table 2 Levels of *BrMYC2/3/4* transcripts

Transgenic lines	max OD	fold
Line1-Ctrl-AVE	18.99	/
BrMYC2-3^{OE}	163.51	8.61
BrMYC2-5^{OE}	210.13	11.07
BrMYC2-6^{OE}	208.41	10.97
Line 2-Ctrl-AVE	23.91	/
BrMYC3-1-2^{OE}	179.41	7.50
BrMYC3-1-5^{OE}	136.82	5.72
BrMYC3-1-7^{OE}	165.31	6.91
BrMYC3-2-1^{OE}	101.64	4.25
BrMYC3-2-7^{OE}	71.13	2.97
BrMYC3-2-9^{OE}	105.87	4.43
Line 3-Ctrl-AVE	20.83	/
BrMYC4-1-1^{OE}	194.97	9.32
BrMYC4-1-4^{OE}	219.54	10.49
BrMYC4-1-5^{OE}	216.56	10.35
BrMYC4-2-1^{OE}	236.97	11.32
BrMYC4-2-4^{OE}	239.28	11.43
BrMYC4-2-5^{OE}	238.69	11.40
